# Lack of Serological and Molecular Association between *Toxoplasma Gondii* Exposure and Obesity: A Case-Control Study

**Published:** 2017-06

**Authors:** Cosme Alvarado-Esquivel, Edith Maldonado-Soto, Luis Francisco Sanchez-Anguiano, Jesus Hernandez-Tinoco, Agar Ramos-Nevarez, Sandra Margarita Cerrillo-Soto, Ada Agustina Sandoval-Carrilo, Jose Manuel Salas-Pacheco, Elizabeth Irasema Antuna-Salcido, Sergio Estrada-Martinez, Carlos Alberto Guido-Arreola

**Affiliations:** 1 Faculty of Medicine and Nutrition, Juarez University of Durango State, Avenida Universidad S/N, 34000 Durango, Dgo, Mexico;; 2 Institute for Scientific Research “Dr. Roberto Rivera Damm”, Juarez University of Durango State, Avenida Universidad S/N, 34000 Durango, Durango. Mexico;; 3 Clinica de Medicina Familiar, Instituto de Seguridad y Servicios Sociales de los Trabajadores del Estado, Predio Canoas S/N, 34079 Durango, Mexico

**Keywords:** Toxoplasma gondii, seroprevalence, obesity, case-control study

## Abstract

The association between *T. gondii* infection and obesity has been scantly studied. Through an age-, and gender-matched case-control study, we determined the association of *T. gondii *infection and obesity using serological and molecular methods. Cases included 203 persons with obesity, and controls included 203 persons without obesity. Participants were tested for the presence of anti-*Toxoplasma* IgG antibodies using an enzyme-linked immunoassay (EIA). IgG seropositive individuals were further tested for the presence of anti-*T. gondii* IgM antibodies using an EIA, and *T. gondii* DNA by polymerase chain reaction (PCR). Anti-*T. gondii* IgG antibodies were found in 16 (7.9%) of the 203 cases and in 18 (8.9%) of the 203 controls (OR=0.87; 95% CI: 0.43-1.77; *P*=0.72). One (6.3%) of the 16 anti-*T. gondii *IgG seropositive cases and 6 (33.3%) of the 18 anti-*T. gondii *IgG seropositive controls were positive for IgM (*P*=0.09). Mean body mass index (35.5 ± 4.5) in *T. gondii* seropositive cases was similar (*P*=0.57) to that (36.1 ± 4.5) found in *T. gondii* seronegative cases. Stratification by obesity classes (I, II, and III) did not reveal differences (*P*>0.05) in seroprevalences (7.8%, 7.9%, and 8.1%, respectively) or high (>150 IU/ml) IgG antibody levels (3.3%, 3.9%, and 2.7%, respectively). PCR was positive in 5 (31.3%) of 16 cases, and in 5 (27.8%) of 18 controls examined (*P*=1.0). We found no serological or molecular evidence of an association between *T. gondii* infection and obesity in people attending a public health center in the northern Mexican city of Durango.

## INTRODUCTION

The coccidian parasite *Toxoplasma gondii* (*T. gondii*) is a common pathogen with worldwide distribution (1). This parasite is usually transmitted to humans by ingestion of food or water contaminated with oocysts shed by cats, and ingestion of raw or undercooked meat containing tissue cysts (2, 3). In addition, primary infection with *T. gondii* in pregnant women may lead to vertical transmission with risk for congenital disease (4). Infection with *T. gondii* is usually asymptomatic, however, this infection may lead to disease of the central nervous system, eyes and lymph nodes (5, 6). In immunocompromised individuals, a reactivation of *T. gondii* infection may result in a severe and life-threatening disease with involvement of the central nervous system (2, 7). Infection with *T. gondii* has been associated with changes in behavior in humans and animals (8, 9). An increase in dopamine production induced by *T. gondii* may contribute to behavioral changes (8). Several psychiatric disorders have been linked to infection with *T. gondii* including schizophrenia (10), bipolar disorder, and obsessive-compulsive disorder (11).

Obesity is a major health problem, and its prevalence is high in many parts of the world (12). In Mexico, more than 50% of adults have overweigh and obesity (13). Obesity and overweight have been linked to important causes of mortality in Mexico including coronary heart disease, type-2 diabetes mellitus, cancer, and stroke (13). Infection with *T. gondii* might be associated with obesity because this infection is usually acquired by food. Obese people may eat in a higher quantity than non-obese people; and therefore, this increase in eating might increase the risk of consuming food likely contaminated with *T. gondii*. It is possible that consumption of a double portion of meat (a well-known source of *T. gondii*) might increase two-fold the risk for acquiring infection. It is also possible that drinking untreated water or consuming unwashed raw vegetables or fruits in high quantities might also increase the risk for *T. gondii* infection. The association of *T. gondii* infection and obesity has been scantly studied. Reeves and coworkers (14) found an association between positive serology to *T. gondii* and obesity in psychiatrically healthy adults. Rubicz and coworkers (15) found a 9% seroprevalence of *T. gondii* infection in Mexican Americans from San Antonio, Texas that suffered from high rates of obesity and type-2 diabetes. In contrast, in a multinational epidemiological study of individuals from Iceland, Sweden and Estonia, no association of anti-*T. gondii* IgG antibodies and body mass index was found (16). However, in a recent study in Germany a body mass index ≥30 was an independent risk factor for IgG seropositivity to *T. gondii* (17). The present study therefore aimed to determine whether *T. gondii* infection is associated with obesity in adults attending a public clinic of family medicine in Durango City, Mexico. Determining this association may help for an optimal planning of preventive measures against *T. gondii* infection.

## METHODS

### Selection and description of participants

Through an age- and gender-matched case control study design, we studied 203 individuals with obesity and 203 individuals without obesity attended in a public clinic of familiar medicine in Durango City, Mexico. This study was performed from June 2015 to August 2016. Inclusion criteria for enrollment of cases were: 1) individuals with obesity attending a public primary health care center (Clinic of Family Medicine, Institute of Security and Social Services of State Workers) in Durango City, Mexico; 2) aged 18 years and older; and 3) who accepted to participate in the study. Socioeconomic status and occupation were not restrictive criteria for enrollment.

Obesity was defined as a body mass index ≥30; and classified in class I, class II, and class III when body mass indexes were 30-34.9, 35.0-39.9, and ≥40.0, respectively (13). Control subjects were matched with cases for age and gender. Cases included 42 (20.7%) males and 161 (79.3%) females, and their mean age was 51.4 ± 11.6 (range 22-83) years old. Controls were randomly selected. Inclusion criteria for enrollment of controls subjects were: 1) individuals without obesity attending the same public primary health care center where cases were selected; 2) aged 18 years and older; and 3) who accepted to participate in the study. Controls included 42 (20.7%) males and 161 (79.3%) females. Mean age in control subjects were 51.5 ± 11.5 (range 20-80) years old. No statistically significant difference (*P*=0.89) in age between cases and controls was found.

### Technical information

Sera from cases and controls were obtained and kept frozen at -20°C until analyzed. Anti-*T. gondii* IgG antibodies were detected in sera using the commercially available enzyme immunoassay (EIA) kit “*Toxoplasma* IgG” (Diagnostic Automation/Cortez Diagnostics Inc., Woodland Hills, CA, USA). Anti-*T. gondii* IgG antibody levels were expressed as International Units (IU)/ml. We used a cut-off of 8 IU/ml for seropositivity. All serum samples positive for anti-*T. gondii* IgG antibodies were further analyzed for anti-*T. gondii* IgM antibodies using the commercially available EIA “*Toxoplasma* IgM” kit (Diagnostic Automation/Cortez Diagnostics Inc.). Both IgG and IgM EIAs were performed following the manufacturer’s instructions.

Cases and controls seropositive for *Toxoplasma*-specific IgG antibodies by EIA were further analyzed to detect DNA of *T. gondii* by nested-polymerase chain reaction (PCR). DNA was extracted from whole blood samples of cases and controls according to a protocol described by Iranpour and Esmailizadeh [http://www.protocol-online.org/prot/Protocols/Rapid-Extraction-of-High-Quality-DNA-from-Whole-Blood-Stored-at-4-C-for-Long-Period-4175.html]. PCR amplification was performed following the PCR protocol described by Roth *et al* (18). Primers directed against the B1 gene of *T. gondii* were used. PCR amplified material was analyzed by agarose gel electrophoresis, stained with ethidium bromide, and visualized by ultraviolet illumination.

### Statistics

Data was analyzed using the software Epi Info 7 and SPSS 15.0 (SPSS Inc. Chicago, Illinois). We calculated the sample size using the following values: a 95% confidence level, a power of 80%, a 1:1 proportion of cases and controls, and a reference seroprevalence of 6.1% (19) as the expected frequency of exposure in controls, and an odds ratio of 2.8. Thus, a sample size of 195 cases and 195 controls was obtained. Age among cases and controls was compared with the student’s *t* test. The association of *T. gondii* infection and obesity was analyzed with the two-tailed Pearson’s chi-squared test. We calculated the odds ratio (OR) and 95% confidence interval (CI), and statistical significance was set at a *P* value < 0.05.

### Ethics aspects

The ethics committee of the Institute of Security and Social Services of State Workers in Durango City, Mexico approved this study. Participation in the study was voluntary, and a written informed consent was obtained from each participant.

## RESULTS

Anti-*T. gondii* IgG antibodies were found in 16 (7.9%) of the 203 cases and in 18 (8.9%) of the 203 controls. The seroprevalence of *T. gondii* infection in cases was similar to the one in controls (OR=0.87; 95% CI: 0.43-1.77; *P*=0.72). Of the 16 anti-*T. gondii* IgG positive cases, 7 (43.8%) had IgG levels higher than 150 IU/ml, one (6.3%) between 100-150 IU/ml, and 8 (50.0%) between 8 to 99 IU/ml. Whereas, of the 18 anti-*T. gondii* IgG positive controls, 13 (72.2%) had IgG levels higher than 150 IU/ml, and 5 (27.8%) between 8 to 99 IU/ml. The frequency of high (>150 IU/ml) anti-*T. gondii* IgG levels in cases was similar to the one in controls (OR=0.29; 95% CI: 0.07-1.24; *P*=0.18). One (6.3%) of the 16 anti-*T. gondii *IgG seropositive cases was positive to anti-*T. gondii* IgM antibodies by EIA. In contrast, 6 (33.3%) of the 18 anti-*T. gondii *IgG seropositive controls were positive to IgM by EIA. No difference in the frequencies of anti-*T. gondii* IgM antibodies among cases and controls was found (*P*=0.09).

Mean body mass index in *T. gondii* seropositive cases (35.5 ± 4.5) was similar (*P*=0.57) to that (36.1 ± 4.5) found in *T. gondii* seronegative cases. Stratification by obesity classes I, II, and III did not show differences (*P*>0.05) in seroprevalences (7.8%, 7.9%, and 8.1%, respectively) or frequency of high IgG antibody levels (3.3%, 3.9%, and 2.7%, respectively).

With respect to detection of *T. gondii* DNA in whole blood of anti-*T. gondii* IgG positive participants, PCR was positive in 5 (31.3%) of 16 cases and in 5 (27.8%) of 18 controls examined. No statistically significant difference in the frequencies of *T. gondii* DNA positivity among cases and controls was found (*P*=1.0). Stratification by age and gender groups did not show differences (*P*>0.05) in seroprevalences among cases and controls (Table 1). *T. gondii* DNA was found in three cases with >150 IU/ml of IgG antibodies and in two cases with <100 IU/ml of IgG antibodies. All 5 cases with *T. gondii* DNA were negative to anti-*T. gondii* IgM antibodies. *T. gondii* DNA was found in three cases with obesity class I, in one case with obesity class II, and in one case with obesity class III.

**Table 1:** Correlation of *T. gondii* seropositivity and demographic variables in cases and controls

Variable	Cases			Controls			*P*. value
	No. of subjects	Seropositive to T. gondii		No. of subjects	Seropositive to T. gondii		
	tested	No.	%	tested	No.	%	

Ages (years)							
30 or less	12	0	0.0	10	1	10.0	0.45
31-50	76	5	6.6	73	7	9.6	0.55
>50	115	11	9.6	120	10	8.3	0.82
Gender							
Female	161	12	7.5	161	9	5.6	0.49
Male	42	4	9.5	42	9	21.4	0.13

## DISCUSSION

Very little is known about the association of *T. gondii* infection and obesity. Results of a few studies about this association have shown conflicting results (14-17). Positive association between seroprevalence of *T. gondii* infection and obesity has been found in adults in Germany (14, 17). In contrast, a low (9%) seroprevalence of *T. gondii* infection in Mexican Americans from San Antonio, Texas that suffered from high rates of obesity and type-2 diabetes was found (15). In addition, no association of seroprevalence of *T. gondii* infection and body mass index was found in a multinational epidemiological study in Iceland, Sweden and Estonia, (16). Therefore, we sought to determine whether *T. gondii* infection is associated with obesity in adults attending a public clinic of family medicine in the northern Mexican city of Durango. For this purpose, we assessed not only the prevalence of anti-*T. gondii* IgG antibodies but also the IgG levels, anti-*T. gondii* IgM seropositivity, and detection of *T. gondii* DNA. Results of the present study indicate that anti-*T gondii* IgG and IgM seropositivity rates, IgG levels, and frequency of *T. gondii* DNA in obese people are similar to those observed in age- and gender-matched control subjects without obesity attended in the same clinic of family medicine. Therefore, our results based on serological and molecular methods do not support an association between obesity and *T. gondii* infection. Results of the present study agree with the lack of association between body mass index and *T. gondii* IgG seroprevalence found in a multinational epidemiological study of individuals from Iceland, Sweden and Estonia (16), and with the low (9%) seroprevalence of *T. gondii* infection found in Mexican Americans that suffered from high rates of obesity and type-2 diabetes reported by Rubicz and coworkers (15). In contrast, our results conflict with those reported in two German studies (14, 17). A positive serology to *T. gondii* associated with obesity in psychiatrically healthy adults in Germany was reported by Reeves and coworkers (14). Furthermore, a body mass index ≥30 was an independent risk factor for IgG seropositivity to *T. gondii* in a nationwide representative cross-sectional study in Germany (17). It is not clear why the association of *T. gondii* infection and obesity was found in populations in Germany but not in obese people in the present study. It is likely that differences in the characteristics of the populations and study designs among the studies might explain the differences in the association. In the study of Reeves and coworkers (14), the association between *T. gondii* infection and obesity was observed in subjects 60 years and older but not in subjects younger than 60 years. Stratification by age groups in our study (<30, 31-50, and >50 years) did not show an association of infection and obesity. In addition, we used an age- and gender-matched case-control study design whereas Reeves and coworkers performed adjustment by age but not by gender. The number of obese participants in the study by Reeves and coworkers was 74 (14), whereas we studied 203 obese participants. On the other hand, in the study of Wilking and coworkers (17) who reported an association of *T. gondii* seropositivity and obesity, researchers studied a large number (1,023) of obese participants, but the study design was cross-sectional, and no adjustment or stratification by age for *T. gondii* seropositivity was performed. In addition, Wilking and coworkers (17) used an automatic enzyme-linked fluorescence assay for detection of anti-*T. gondii* IgG antibodies whereas we used a manual enzyme-linked immunosorbent assay. We are not aware of a previous study about the association of *T. gondii* infection and obesity using molecular methods. However, in the present study using *T. gondii* PCR, no association between obesity and *T. gondii* DNA was found. The lack of association between obesity and *T. gondii* was unexpected since obese people may eat more than non-obese people; and therefore, an increase in eating might increase the risk of consuming food likely contaminated with *T. gondii*.

The limitations of the present study include the investigation of a relatively small cohort of obese people attending a single public health center. The socioeconomic status of people attending the participating health center are mostly medium, and it is not clear whether the association of obesity and *T. gondii* infection might occur in people of low or high socioeconomic status.

## CONCLUSIONS

We conclude that there is not serological or molecular evidence of an association between *T. gondii* infection and obesity in people attended in a public family medicine health center in the northern Mexican city of Durango. Further research to elucidate the role of *T. gondii* in obesity is needed.

## ABBREVIATIONS

CI: Confidence interval
EIA: Enzyme immunoassay
IU: International units
OR: Odds ratio
PCR: Polymerase chain reaction
